# Variation of Fungal Metabolites in Sorghum Malts Used to Prepare Namibian Traditional Fermented Beverages *Omalodu* and *Otombo*

**DOI:** 10.3390/toxins11030165

**Published:** 2019-03-16

**Authors:** Sylvia N. Nafuka, Jane M. Misihairabgwi, Ronnie Bock, Anthony Ishola, Michael Sulyok, Rudolf Krska

**Affiliations:** 1 Department of Biological Sciences, Faculty of Science, University of Namibia, Windhoek 10005, Namibia; rbock@unam.na; 2 Department of Biochemistry and Microbiology, School of Medicine, University of Namibia, Windhoek 10005, Namibia; jmisihairabgwi@unam.na; 3 Department of Pharmaceutical Chemistry and Phytochemistry, School of Pharmacy, University of Namibia, Windhoek 10005, Namibia; aishola@unam.na; 4 Center for Analytical Chemistry, Department of Agro Biotechnology (IFA-Tulln), University of Natural Resources and Life Sciences Vienna (BOKU), Konrad Lorenz Str. 20, 3430 Tulln, Austria; michael.sulyok@boku.ac.at (M.S.); rudolf.krska@boku.ac.at (R.K.); 5 Institute for Global Food Security, School of Biological Sciences, Queen’s University Belfast, University Road, Belfast BT7 1NN, Northern Ireland, UK

**Keywords:** traditional sorghum malts, mycotoxins, aflatoxins, *Aspergillus*, LC/MS/MS

## Abstract

Sorghum malts, which are important ingredients in traditional fermented beverages, are commonly infected by mycotoxigenic fungi and mycotoxins may transfer into the beverages, risking consumers’ health. Liquid chromatography–tandem mass spectrometry was used to determine variation of fungal metabolites in 81 sorghum malts processed for brewing of Namibian beverages, *otombo* (*n* = 45) and *omalodu* (*n* = 36). Co-occurrence of European Union (EU)-regulated mycotoxins, such as patulin, aflatoxins (B_1_, B_2_, and G_2_), and fumonisins (B_1_, B_2_, and B_3_) was detected in both malts with a prevalence range of 2–84%. Aflatoxin B_1_ was quantified in *omalodu* (44%) and *otombo* malts (14%), with 20% of *omalodu* malts and 40% of *otombo* malts having levels above the EU allowable limit. Fumonisin B_1_ was quantified in both *omalodu* (84%) and *otombo* (42%) malts. Emerging mycotoxins, aflatoxin precursors, and ergot alkaloids were quantified in both malts. Notably, 102 metabolites were quantified in both malts, with 96% in *omalodu* malts and 93% in *otombo* malts. An average of 48 metabolites were quantified in *otombo* malts while an average of 67 metabolites were quantified in *omalodu* malts. The study accentuates the need to monitor mycotoxins in sorghum malts intended for brewing and to determine their fate in the beverages.

## 1. Introduction

*Sorghum* is a genus of cereals in the family Poaceae of approximately 30 species. One species, *Sorghum bicolor*, is native to Africa and is the world’s fifth most important cultivated cereal crop [1], with many significant uses, such as being a staple food in some sub-Saharan countries, the main ingredient in the production of beverages, and animal feed [2]. In Namibia, sorghum is mainly cultivated by rural subsistence farmers of the northern regions and is mainly used for the brewing of traditional beverages [3]. Specifically, unground malted sorghum grains are used as the main ingredients in the brewing of the traditional alcoholic drink locally known as *otombo*, while malted sorghum flour is used for the brewing of the non-alcoholic beverages *omalodu* and oshikundu [3]. *Otombo*, which is mainly brewed nationwide for income generation, is sold at shebeens in rural areas and at open markets in urban areas, and, due to its alcoholic content, is generally consumed by elders. *Omalodu* is also a popular ceremonial traditional beverage in Namibian Oshiwambo and Rukwangali communities. In both communities, *omalodu* is primarily consumed at sociocultural ceremonies.

Sorghum malts used in this study vary depending on the malting process and the milling stages. According to [4], malting is defined as the germination of grains to promote the development of hydrolytic enzymes which were inactive in the raw grain. Generally, the malting process involves three main processes: Steeping, germination, and drying [5]. In Namibia, particularly among the Oshiwambo and Rukwangali communities, the malting process is carried out at the household level and is similar, with minor differences due to cultural specifications and weather conditions. Basically, the process of sorghum malting involves steeping the cleaned grains in water for 24 to 48 h, draining, and germination in sealed plastics, jute sacks, or metal trays for 1 to 2 weeks. Some Oshiwambo community members may add sandy soil to facilitate the germination process. The germinated grains are then air dried at ambient temperature, then the dry malted grains, including the root fragments, are used for *otombo* brewing. Milling of the dry malts used for *omalodu* brewing is usually carried out in a hut or an open area by pounding with strong wooden sticks in a wooden traditional mill. The pounding is continued until all grains are pulverized with intermittent sifting using a circular basket made from palm leaves. The initial round of sifted coarse sorghum flour with grains and root fragments is reserved for *omalodu* brewing. *Omalodu* malts are mainly prepared for brewing at the household level and for family use, while some may also be transported and sold at open markets in urban areas. *Otombo* malts are prepared for brewing at shebeens and for selling at open markets.

Due to the warm, moist, and likely unhygienic conditions during the traditional malting and milling processes, the growth of mycotoxigenic fungi is stimulated [6]. In addition, mycotoxigenic fungi can infiltrate deep into sorghum matrices and produce mycotoxins during the pre-harvest, storage, transportation, processing, and marketing stages [7]. Mycotoxins are fungal secondary metabolites representing natural contaminants in raw materials, foods, and feeds [8]. The most dangerous mycotoxins are aflatoxins, ochratoxins, fumonisins, patulin, and ergot alkaloids, produced by fungi belonging to *Aspergillus*, *Penicillium*, *Claviceps*, and *Fusarium* genera [9]. The toxins are known to have carcinogenic, mutagenic, teratogenic, cytotoxic, neurotoxic, nephrotoxic, estrogenic, dermotoxic, and immunotoxic effects in humans [10,11]. Many parts of the world are regulating mycotoxins by creating maximum allowable limits in different foods and feed. According to a global food prevalence mycotoxin survey by [12], 72% of the food samples, including cereals, contained detectable amounts of mycotoxins addressed by regulatory limits in the European Union (EU). However, other fungal metabolites, such as beauvericin (BEA), moniliformin (MON), sterigmatocystin (STE), emodin (EMO), alternariol (AOH), tenuazonic acid (TeA), and 3-Nitropropionic acid, (3-NPA), are now frequently quantified in a variety of foods and feed in different parts of the world [13]. There are also indications that the incidence of these so-called emerging mycotoxins, which are neither routinely determined nor legislatively regulated, is rapidly increasing [14,15,16].

The quality of raw materials used to prepare traditional beverages influences the final product safety. Many reports on the occurrence and quantities of fungal metabolites in sorghum malts and related products from countries neighboring Namibia have been documented in South Africa [17], Zimbabwe [18], and Botswana [19]. A previous study in Namibia, which determined the diversity of fungal metabolites in sorghum malt samples used for oshikundu beverage production and their transfer rates into the beverage, reported that, although EU-legislated mycotoxins were not quantifiable in the beverage, transfer rates into the beverage were above 50% for most of the other fungal metabolites [20]. Due to the diversity of malting and processing methods for different beverages, the types and quantities of fungal metabolites in the specific malts may vary. Consequently, consumers’ exposure to the metabolites will also vary. There is, therefore, a need to investigate the quality of raw materials used to prepare other traditional Namibian beverages, such as *otombo* and *omalodu*, which are consumed daily and by many people. This study, therefore, aimed at determining the occurrence and variation of fungal metabolites in sorghum malts intended for the brewing of *omalodu* and *otombo* beverages.

## 2. Results and Discussion

### 2.1. Occurrence of Fungal Metabolites in Sorghum Malts for Omalodu and Otombo Brewing

Only metabolite concentration levels that were above the limits of quantification (LOQ) were quantified for both malts. Hence, a total of 102 fungal and bacterial metabolites, including seven regulated mycotoxins, were quantified in sorghum malts for both *omalodu* and *otombo* beverages (Table 1, Table 2 and Table 3). Only 4% of the 102 metabolites were of non-fungal origin. The occurrence of fungal metabolites, including mycotoxins in sorghum malts intended for the beverage oshikundu, quantified using the same analytical technique and method, was previously reported in Namibia [20], with a total of 98 metabolites quantified. Other reports on the occurrence and quantities of fungal metabolites in sorghum malts and grains from Southern Africa have been documented in South Africa [17], Malawi [21], Zimbabwe [18], and Botswana [19].

The total number of metabolites was higher in *omalodu* malts (*n* = 101) than in *otombo* malts (*n* = 96). An average of 48 metabolites was quantified in *otombo* malts, while an average of 67 metabolites was quantified in *omalodu* malts, respectively. Although some metabolites were quantified with low prevalence rates and quantities, the risks of exposure to these complex mixtures of metabolites by consumption of brews should be studied in more detail in order to minimize the possible synergistic and/or additive effects during brewing.

The metabolites detected were representatives of the following mycotoxigenic fungal genera: *Aspergillus* 34%, (Table 1 and Table 2) *Penicillium* 16%, (Table 1), *Fusarium* 15%, (Table 1 and Table 2), *Alternaria* 7% (Table 1 and Table 2), and *Claviceps* 6% (Table 1), while 22% were non-fungal metabolites or produced by unspecified and uncommon fungal genera (Table 3). According to [22], these genera are known to be associated with sorghum malts and grains in Nigeria, Botswana [19], and Ethiopia [23] and also known as the main mycotoxigenic fungal genera [24,25]. Only 2% of the metabolites were 100% prevalent in *otombo* malts, whereas 15% of the metabolites were 100% prevalent in *omalodu* malts. Of the quantified fungal metabolites, 64%, 69%, 65%, and 85% of the *Aspergillus*, *Fusarium*, *Penicillium*, and *Alternaria* metabolites had higher average concentrations in *omalodu* malts than in *otombo* malts, respectively.

### 2.2. Variation of Regulated Mycotoxins and Aflatoxins Precursors

Seven mycotoxins addressed by regulatory limits in the EU (i.e., aflatoxin B_1_, B_2_, and G_1_, fumonisins B_1_, B_2_, and B_3_, and patulin) were quantified in both malts (Table 1). Due to the absence of such limits in Namibia, the limits fixed by the EU [26] were used as the basis for discussion in the present study. The same mycotoxins have been recently reported in sorghum malts from Namibia using the same analytic method by [20], with the exception of patulin found in the present study and fumonisin B_4_ quantified only in the previous study. Overall, 98% and 76% of the *omalodu* and *otombo* samples were contaminated with at least one of the EU regulated toxins, respectively. Comparing the two groups of malt samples, 71% of the of the EU-regulated mycotoxins had higher incidences in *omalodu* malts than in *otombo* malts, while 47% of these mycotoxins had higher average concentrations in *otombo* malts than in *omalodu* malts.

AFB_1_ was quantified in *omalodu* (prevalence = 44%, average = 2.87 ± 2.93 µg/kg) and *otombo* malts (prevalence = 14%, average = 15.1 ± 22.9 µg/kg) with 20% of *omalodu* malts and 40% of *otombo* malts having levels above the EU regulatory limit of 5 µg/kg. An independent-samples *t*-test conducted to compare average AFB_1_ levels in *omalodu* and *otombo* malts showed that the difference was not statistically significant (*p* > 0.05). Differences in averages of AFB_2_, AFG_1_, FB_1_, FB_2_, and FB_3_ levels between *omalodu* and *otombo* malts were also not statistically significant (*p* > 0.05). These results suggest that the different malt preparation methods may not have a significant effect on levels of aflatoxins. In the previous study [20], AFB_1_ was quantified with higher prevalence rate (50%) and level (average: 4.5 ± 5.5 µg/kg) in the sorghum malt flour samples compared with *omalodu* malts (44% prevalence; average: 2.87 ± 2.93 µg/kg).

Other reports regarding aflatoxin occurrence in sorghum malt grain samples intended for beverage production in Africa analyzed using other techniques were conducted: In Malawi [21], total aflatoxin contents were identified via immunoaffinity column and were reportedly higher (408 ± 68 µg/kg) in sorghum malts prepared for beer brewing than in the current study (5.47 ± 13.8 µg/kg). In Burkina Faso [27], aflatoxin B_1_ and ochratoxin A were purified with immunoaffinity columns and analyzed using high-performance liquid chromatography (HPLC), revealing a higher AFB_1_ level (97.6 ± 88.2 µg/kg) for malt samples than the present study (8.49 ± 16.9 µg/kg). In South Africa [17], mycotoxins were identified using a multi-mycotoxin thin-layer chromatography method and quantified via HPLC to screen sorghum malt grains intended for traditional beers (Utshwala). The former study revealed the presence of zearalenone and absence of AFB_1_ in sorghum malt grains, while the current study revealed contrasting results. FB_1_ is one of the common toxicologically important mycotoxins and was quantified in *omalodu* malts with 84% prevalence (average concentration: 61.4 ± 70 µg/kg) and in *otombo* malts with a prevalence of 42% (average concentration: 29.1 ± 25.7 µg/kg) (Table 1).

FB_1_ was quantified with a higher prevalence rate (75%) in sorghum flour malts from Namibia prepared for oshikundu beverage [20] compared to the current study. In Botswana, FB_1_ was detected at a lower prevalence rate of 6% in sorghum malt samples, with concentrations ranging from 47 to 1316 µg/kg [19]. All sorghum malt samples had FB_1_ concentrations below the EU regulatory level of 2000 µg/kg.

Patulin, which is normally found in fruits and vegetables, particularly apple and its products [28,29,30], was quantified in a single sample of *omalodu* (average = 57.7 µg/kg) and only two samples of *otombo* malts (average = 183.1 ± 143.2 µg/kg). However, the average concentrations for *omalodu* and *otombo* malts were higher than those fixed by the EU at 50 µg/kg for patulin in apple juice. Patulin is of concern because it is produced by many fungal genera and is suspected of being clastogenic, mutagenic, teratogenic, genotoxic, and cytotoxic [31]. The co-occurrence of different regulated toxins suggests synergistic toxic effects that raise concerns on the health hazards associated with these malts.

Several metabolites from the biosynthetic pathway of aflatoxins, namely averufanin, averufin, STE, and versicolorin C (Table 1), were quantified in the sorghum malts. The same aflatoxin precursors were reported by [20] in sorghum malts prepared for oshikundu, with the exception of versicolorin C, found only in the present study. Averufanin was quantified in 27% of the *omalodu* malts and not in the *otombo* malts, while averufin was quantified in 84% of the *otombo* malts but not in *omalodu* malts. A high incidence rate of 91% (average = 95.49 µg/kg) was recorded for STE from *omalodu* malts. STE is classified as a possible human carcinogen by the International Agency of Research in Cancer [32]. In addition, in vitro genotoxic and cytotoxic studies of STE revealed that it is genotoxic to liver hepatocellular cells [33] and cytotoxic to immortalized ovarian hamster cells [34] and liver hepatocellular cells [35].

### 2.3. Emerging Mycotoxins and Ergot Alkaloids Quantified in Sorghum Malts

As shown in Table 1, some emerging mycotoxins, especially 3-NPA, EMO, AOH, AME, TeA, MON, STE, and BEA, occurred with prevalence ranges of 84–100% in *omalodu* malts and 17–100% in *otombo* malts. A magnetic resonance imaging study by [36] reported that 3-NPA is a potent mitochondrial toxin and neurotoxin. 3-NPA was observed in all sorghum flour malt samples, similar to previously reported results in Namibia [20]. The average concentration of 3-NPA was lower (2530 ± 2860 μg/kg) in malts for oshikundu [20] than that observed in malts for *otombo* (3290 ± 5000 µg/kg). MON was not quantified in sorghum flour malts in the previous study [20], but it was observed in all samples of *omalodu* malts and quantified with a concentration of 348 ± 511 μg/kg and with concentration of 75.6 ± 135.9 μg/kg in *otombo* malts. In vivo toxicity investigations suggest that MON can induce cardiotoxicity [37] and cause immunosuppression, muscular weakness, and intestinal problems in poultry [38]. EMO is both a fungal and plant metabolite and it was quantified with average concentrations of 23.7 ± 17.7 μg/kg and 19.6 ± 31.9 μg/kg in *omalodu* and *otombo* malts, respectively. Several studies demonstrated that EMO has anticancer [39,40], antiviral [41,42], and antibacterial activity [43]. BEA was quantified at concentrations of 5.08 ± 7.01 μg/kg and 1.60 ± 1.72 μg/kg in *omalodu* and *otombo* malts, respectively. BEA has shown cytotoxic effects on human cell lines [44]. Among the *Alternaria* toxins, TeA was quantified with high amounts in both *omalodu* (1925.6 ± 3406.4 μg/kg) and *otombo* (999 ± 2170 μg/kg) malts, compared to lower amounts of AOH (14 ± 30 μg/kg) and (9.99 ± 18.09 μg/kg) in *omalodu* and *otombo* malts, respectively. Some in vivo studies of TeA revealed that it is toxic to animals, such as mice and rats [45], beagle dogs, monkeys [46], and chickens [47]. According to an in vitro study [48], AOH and AME are mutagenic to hamster lung fibroblast cells lines.

Six clavine ergot alkaloids, synthesized mainly by fungal species of *Claviceps* genera were quantified in both malt samples. Elymoclavine was observed in 3% and 38% of *otombo* and *omalodu* malt samples, respectively. Other alkaloids were observed with high prevalence (50–100%) in both malt samples (Table 1). Ergot alkaloids are typically important because chronic poisoning by these toxins through consumption of contaminated grain products causes ergotism. Sorghum crops are also vulnerable to ergot disease during cultivation. In Africa, the pathogen is recognized as a distinct species, *Claviceps africana* [49]. Damages caused by *C. africana* have been recognized as a major cause for decreased quality and nutritive value of sorghum grains [50].

### 2.4. Other Fungal Metabolites Quantified in Sorghum Malts

The prevalence and concentrations of other fungal metabolites quantified in both malt samples are indicated in Table 2. Metabolites produced solely by *Aspergillus* genus were mostly quantified at a prevalence of 49%; 19 of these metabolites were quantified in 80% to 100% of *omalodu* samples analyzed, as opposed to three metabolites of the same prevalence rate quantified in *otombo* malts. Secondary metabolites of *Aspergillus* are representatives from the following groups: Gliotoxins, fumitremorgins, fumagillins and fumiquinazolines, helvolic acids, tryptoquivalines, and pseurotins. Although there are no regulations in force for these metabolites, some of them have their in vitro toxicities reported. An example is gliotoxin, an epipolythiodioxopiperazine produced by *Aspergillus fumigatus* and quantified in both *omalodu* (78%) and *otombo* (44%) malts. Its disulfide bridge may cause immunosuppressive properties and apoptosis in macrophages and monocytes [51]. Bis (methylthio) gliotoxin is an inactive derivative of gliotoxin, proposed as a stable biomarker for invasive aspergillosis [52]. Another toxic metabolite from *Aspergillus flavus* quantified in both *omalodu* (69%) and *otombo* (39%) malts is cyclopiazonic acid, an indole tetramic. Cyclopiazonic acid causes degenerative changes and necrosis in the liver, spleen, pancreas, kidney, salivary glands, myocardium, and skeletal muscles, based on toxic effects observed in male and female rats [53]. Higher prevalence rates of *Aspergillus* metabolites are an indication of the higher contamination by storage mycotoxigenic fungi such as *Aspergillus fumigatus*, *Aspergillus clavatus* and *Aspergillus niger.* The latter findings are expected because both malts are traditionally processed under likely unhygienic conditions. The poor storage conditions for prolonged times at homes and markets makes the malts susceptible to fungal contamination. Other metabolites synthesized by *Fusarium*, *Penicillium*, and *Alternaria* species were quantified as well.

### 2.5. Other Metabolites Quantified in Sorghum Malts

Twenty-two metabolites synthesized by non-fungal organisms and uncommon fungal species were quantified in both *omalodu* and *otombo* malts (Table 3). Dihydroxymellein and tryptophol were absent in *omalodu* malts, while abscisic acid was absent in *otombo* malts. Four diketopiperazines synthesized by fusion of 2 different amino acids, namely Cyclo (l-Pro-l-Tyr), or maculosin, cyclo (l-Pro-l-Val), brevianamide F, or cyclo (l-Trp-l-Pro), Fellutanine A or cyclo (l-Trp-l-Trp), were quantified in both *omalodu* and *otombo* malts. Cyclo (l-Pro-l-Tyr) is formed by the fusion of tyrosine and proline and has been reported as a secondary metabolite of various fungi [54] and bacteria [55]. Additionally, it is identified as a host-specific phytotoxin produced by *Alternaria alternata* [54]. It was quantified at a prevalence of 100% in *omalodu* malts and at 94% in *otombo* and at concentration ranges of 48.2–48,200 μg/kg in *omalodu* and 32.5–165 μg/kg in *otombo* malts, respectively. Cyclo (l-Pro-l-Val) is formed by the fusion of valine and proline and synthesized by marine *Penicillium* species [56]. It was quantified at higher average concentrations (456 ± 158 µg/kg) in *omalodu* malts than in *otombo* malts (121 ± 86.4 µg/kg) and had a maximum prevalence of 100% in both malts. Brevianamide F is the simplest member and the biosynthetic precursor of prenylated tryptophan-proline 2.5-diketopiperazines that are produced mainly by *Aspergillus fumigatus* and other *Aspergillus* species [57]. In addition, Brevianamide F is produced by many *Penicillium* species and intermediaries of many fungal species. The brevianamide F average concentration of these malts was highest in *omalodu* malts (144 ± 92.6 µg/kg) the lowest in *otombo* malts (90.1 ± 56.1 µg/kg). Fellutanine A is bio-active, naturally occurring, 2.5 diketopiperazine alkaloid synthesized by *Penicillium fellutanum* and *Penicillium simplicissimum*, [58]. It is also understood to be a non-annulated analogue of “cis” cyclic dipeptide, cyclo (l-Trp-l-Trp). The concentration range of this metabolite in the samples is indicated in Table 3. Tryptophol is an aromatic alcohol that induces sleep in humans and is produced by the trypanosomal parasite in wine as a secondary product of alcoholic fermentation [59]. Tryptophol is also formed from tryptophan during fermentation as well. *Otombo* malts had the highest concentration of 110 ± 80.5 µg/kg, but it was not quantified in *omalodu* malts.

### 2.6. Method Performance Characteristics

The values reported in Table 4 are for the LC/MS/MS method validation characteristics, such as limits of detection (LOD) and LOQ, apparent recoveries (i.e., spiked samples vs. solvent standards), and relative standard deviations (RSD). LOD were observed from 0.02 to 124 ng/g while LOQ were observed from 0.03 to 421 ng/g. Deviations from the target range of 50–120% of apparent recoveries, set by the Commission Regulation (EC) No 401/2006, are mainly caused by matrix effects, whereas the recovery of the extraction step has been determined to be in this target range for the majority of all investigated compounds in other matrices (manuscript in preparation). In addition, the determination of the apparent recoveries was hampered by the fact that 15% of the metabolites (e.g., 3-nitropropionic acid, kojic acid) none of the samples were true blanks. This resulted in apparent recoveries significantly larger than 120%, despite a correction for the concentration in the blank samples being performed. The same holds true for large values for the respective RSD not complying with the <20% criterion that are set for replicate analysis, whereas in this study, three different individual samples were spiked, which potentially resulted in higher values for the repeatability and the combined method uncertainty [60].

## 3. Conclusions

The present study reports data on the variation of fungal metabolites in two different sorghum malts as raw materials for the brewing of two indigenous and popular traditional Namibian beverages, *otombo* and *omalodu*. Both malts were substantially contaminated with fungal metabolites produced by major mycotoxigenic fungal genera. The study found little contamination variation between the two malts. Regulated mycotoxins, emerging mycotoxins, aflatoxin precursors, and ergot alkaloids were quantified in both malts. Generally, the study findings were that *omalodu* malts were mostly contaminated with fungal metabolites and health risk mycotoxin groups than *otombo* malts. Based on the high incidence of mycotoxins and other metabolites in both malt samples, adequate milling and processing conditions (low moisture) must be ensured to reduce the prevalence of these toxins. The present study on two sorghum malts provides three major findings: First is the co-occurrence of seven EU-regulated mycotoxins in both malts, particularly toxic AFB_1_, which was quantified in 20% of *omalodu* malts and 40% of *otombo* malts at levels above the EU allowable limit of 5 μg/kg. Second is the high occurrence of several fungal metabolites in both malts and the existing knowledge gap on the effects of such intricate metabolite mixtures in humans. The third is the high incidence of emerging mycotoxins such as 3-NPA, MON, STE, and TeA and pending risk assessment studies for these toxins in humans. Since traditional malting and processing are likely carried out by mycotoxin-unaware traditional processors, it is also advised to educate the public on the health risks of mycotoxins and possible methods to alleviate fungal contamination and on hygienic conditions during malting and storage. Data from the present study serves as a foundation for more detailed mycotoxin-related studies, such as further investigation on the fate of mycotoxins during the brewing processes of these beverages, considering the possible formation of masked/bound mycotoxins which may not have been quantifiable in the present study. Investigations of the occurrence of fungal metabolites in other indigenous food commodities from Namibia are necessary, as well as the determination of exposure to mycotoxins and their health effects in the Namibian population.

## 4. Materials and Methods

### 4.1. Sorghum Malts Collection

A total of 81 sorghum malt samples, purchased in November 2017 at open-markets in Oshana region, Namibia, were collected for this study. The sorghum malt samples were purchased based on availability at the open markets, hence, 45 sorghum flour malt samples intended for *omalodu* brewing and 36 un-milled sorghum malts grain samples intended for *otombo* brewing. Approximately, 500 g of the samples were collected following the sampling procedure described by [61]. Samples were placed in paper bags, transported to the Centre for Analytical Chemistry, Department of Agrobiotechnology, (IFA-Tulln), University of Natural Resources and Life Sciences, Austria, and stored at −20 °C until analysis.

### 4.2. Metabolites Extraction and Analysis by LC/MS/MS

Sorghum malt samples were extracted for the presence of targeted multi-metabolites, including regulated, conjugated, and emerging mycotoxins. The extraction was done according to the methods described by [26]. Briefly, 5 g of each milled sample and 20 mL of acetonitrile/water/acetic acid (79:20:1, *v*/*v*/*v*) was agitated in a 50 mL polypropylene tube for 90 min at 180 rpm using a rotary shaker (GFL 3017, Burgwedel, Germany). The mixture was then settled and supernatants/extracts were stored at −20 °C until further analysis.

The occurrences of fungal metabolites were detected and quantified using the procedure described by [62]. Briefly, 500 μL of each extract was diluted with an equal volume of acetonitrile/water/acetic acid (79:20:1, *v*/*v*/*v*) and 5 µL was directly injected into the LC/MS/MS system consisting of an Agilent (Waldbronn, Germany) 1290 HPLC and an AB Sciex 5500 QTrap MS/MS with an electrospray ionization (ESI) triple quadrupole. Chromatographic separation was performed on a Phenomenex Gemini C18 column (150 × 4.6 mm, 5 µm) equipped with a C18 (4 × 3 mm) i.d. security guard cartridge, eluted with a gradient of methanol/water containing ammonium acetate and acetic acid.

Data acquisition was achieved in the time-scheduled multiple reactions monitoring (MRM) mode both in positive and negative polarities in two separate chromatographic runs per sample. The expected retention time of the MRM detection window of each metabolite was set at about 27 s and about 48 s for both positive and negative modes, respectively. Data were analyzed using MultiQuant™ 3.0.3 software (AB Sciex, Foster City, CA, USA). Quantification of metabolites was performed using external calibration based on serial dilution of a multi-metabolites stock solution. Results were corrected for apparent recoveries based on relative responses of the two matrices by spiking three different approximately blank samples at three concentration levels. Limits of detection and limits of quantification were determined following the Eurachem guide described by [63]. The accuracy of the method is verified on a routine basis by participation in interlaboratory testing schemes including a broad variation of matrices of grains, nuts, dried fruits, spices, baby food, and animal feed. Satisfactory z-scores between −2 and 2 have been obtained for >94% of the >1000 results submitted so far and for 11 of the 12 results submitted for sorghum, respectively.

Confirmation of positive metabolite identification was attained by the acquisition of two MRMs per metabolite (apart from moniliformin and 3-nitropropionic acid, which displayed only one fragment ion). This generated 4.0 identification points according to Ref. [63]. In addition, the LC retention time and the intensity ratio of the two MRM transitions agreed with the related values of a true standard within 0.03 min and 30% relatively and singly.

### 4.3. Data Analysis

Data evaluation, averages, and range calculations were performed in Microsoft^®^ Excel 2010. An independent-samples *t*-test was done using the Statistical Package for the Social Sciences (SPSS) software, version 21.0 (SPSS, Inc., Chicago, IL, USA).

## Figures and Tables

**Table 1:** Regulated mycotoxins, aflatoxin precursors, ergot alkaloids, and emerging mycotoxins quantified in sorghum malts for the production of *omalodu* and *otombo* beverages.

Compounds	Types	Origin	*Omalodu* Malts *n* = 45			*Otombo* Malts *n* = 36		
			Prevalence (%)	Range (μg/kg)	Average (μg/kg)	Prevalence (%)	Range (μg/kg)	Average (μg/kg)
Aflatoxin B_1_	Regulated	*Aspergillus*	44	0.61–28.3	2.87 ± 2.93	14	0.56–54.2	15.1 ± 22.9
Aflatoxin B_2_			9	0.14–2.35	0.15 ± 0.44	5	0.5–4.48	2.49 ± 2.8
Aflatoxin G_1_			17	0.39–6.95	1.19 ± 1.10	3	0.4	0.4
Patulin			2	57.7	57.7	6	81.8–284.3	183.1 ± 143.2
Fumonisin B_1_		*Fusarium*	84	12–500.2	61.4 ± 70	42	8.17–88.3	29.12 ± 25.7
Fumonisin B_2_			66	7.55–79.46	17.56 ± 12.1	22	5.92–46.8	16.4 ± 13
Fumonisin B_3_			7	21.6–136.6	60.14 ± 66.3	3	22	22
Averufanin	Aflatoxin precursors	*Aspergillus*	29	13.5–384	37.8 ± 47.3	N/D	N/D	N/D
Averufin			N/D	N/D	N/D	83	0.09–103	6.73 ± 20.2
Versicolorin C			13	89.8–200	1534 ± 33.5	24	29.8–2815	444 ± 846
Sterigmatocystin	Aflatoxin precursor and emerging mycotoxin		89	377–1690	4.30 ± 7.98	17	29.8–2810	6.24 ± 4.53
3-Nitropropionic acid	Emerging mycotoxins		100	83.7–10,200	3290 ± 5000	94	7.61–14,900	2530 ± 2860
Alternariol		*Alternaria*	91	1.24–318	14 ± 30	72	0.45–71.42	9.99 ± 18.09
Alternariolmethylether			84	1.27–564	45.7 ± 90.4	42	1.61–80.2	23.6 ± 26.4
Tenuazonic acid			73	132.4–13,400	1925.6 ± 3406.4	81	4.84–11,400	999 ± 2170
Beauvericin		*Fusarium*	97	0.23–30.4	5.08 ± 7.01	39	0.24–5.65	1.60 ± 1.72
Emodin		Plants and *Fusarium*	84	2.16–79.2	23.7 ± 17.7	97	0.35–93.4	19.6 ± 31.9
Moniliformin		*Fusarium*	100	11.3–1550	348 ± 511	94	4.58–728.2	75.6 ± 135.9
Agroclavine	Ergot alkaloids	*Claviceps*	96	18.7–20,500	733 ± 2760	50	6.7–95.4	43.8 ± 31.3
Chanoclavin			98	0.37–188	46.3 ± 40.7	72	0.39–49.7	10.8 ± 14.9
Elymoclavine			38	0.89–153	10.4 ± 27.4	3	1.48	1.48
Festuclavine			100	25.7–11,400	1690 ± 1750	83	1.23–5660	570 ± 1120
Fumigaclavine A			96	0.004–613	89.4 ± 118	89	0.55–118	20.7 ± 29.7
Fumigaclavine C			100	6.49–6040	1060 ± 1260	86	3.26–1159.2	228.4 ± 332.5

N/D = Not detected.

**Table 2:** Unregulated metabolites quantified in sorghum malt samples for the production of *omalodu* and *otombo* beverages.

Compounds	*Omalodu* Malts *n* = 45			*Otombo* Malts *n* = 36		
	Prevalence (%)	Range [(μg/kg) or Peak Area^2^]	Average [(μg/kg) or Peak Area^2^]	Prevalence (%)	Range [(μg/kg) or Peak Area^2^]	Average [(μg/kg) or Peak Area^2^]
*Aspergillus*						
Asperfuran	96	1980–669,000	46,000 ± 110,000	89	14.8–428,000	60,000 ± 88,000
Asterric acid	22	1.09–170	43.6 ± 104	3	62.8	62.8 ± 0.00
Bis (methylthio) gliotoxin	87	4.77–699	103 ± 128	61	4.07–229.9	34 ± 49.9
Bisdethio (methylthio) gliotoxin	64	6.04–263	77.9 ± 62.9	67	1.14–285	35.4 ± 57.6
Gliotoxin	78	3.55–193.7	54.3 ± 55.7	44	3.12–44.9	13.9 ± 11.4
Cyclopiazonic acid	69	55.17–2070	456.15 ± 652.18	39	60.4–486	122 ± 134
Cytochalasin E	84	1.66–96.7	47.5 ± 96.7	42	2.24–521	74.5 ± 135.6
Deoxynortryptoquivalin	91	2.59–727	67.70 ± 122.94	47	2.15–467	57.6 ± 114
Deoxytryptoquivaline A	87	2.32–894	46.57 ± 116.81	44	1.15–142	30.2 ± 47.9
Dihydrocitrinone	41	2.63–184	21.17 ± 49.4	17	2.98–274	50.7 ± 109.6
Flavoglaucin	89	0.51–949	79 ± 222	69	0.16–2810	306 ± 654
Fumagillin	91	6.76–2220	478.85 ± 814.68	44	36–1910	321.3 ± 465.7
Fumiquinazolin A	89	11–979	267 ± 256	61	3.37–224	85.4 ± 85.4
Fumiquinazolin D	100	5.95–3140	826 ± 745	89	1.06–837	175 ± 237
Fumitremorgin C	89	1.70–1140	142 ± 286	56	0.71–411	51.8 ± 90.7
Trypacidin	11	0.41–20.3	3.74 ± 4.58	ND	ND	ND
Tryprostatin B *	88	259,000–130,000,000	12,100,000 ± 24,000,000	6	17,200–22,400	19,800 ± 3680
Tryptoquivaline A	77	1.62–1040	54.7 ± 178	39	1.49–442	75 ± 125.4
Tryptoquivaline F *	88	356,000–14,300,000	4,250,000 ± 3,220,000	20	80,500–4,710,000	2,240,000 ± 1,440,000
Helvolic acid	87	21.7–2860	696 ± 680	47	16.9–2350	329.6 ± 556.2
Kojic acid	100	631–182,000	41,000 ± 48,000	56	1594–52,296	17,712.5 ± 14,125.8
Nigragillin *	100	244,000–113,000,000	27,800,000 ± 31,200,000	94	112,000–92,900,000	7,310,000 ± 16,500,000
Phenopyrrozin	100	9.43–35.8	805 ± 891	64	10.6–7.14	3.03 ± 1.64
Pseurotin A	91	8.54–4400	805 ± 902	64	10.58–764.3	198.9 ± 216.1
Pseurotin D *	82	24,900–996,000	243,000 ± 199,000	N/D	N/D	N/D
Iso-Rhodoptilometrin	91	0.13–6.91	1.70 ± 1.91	78	0.11–4.28	0.71 ± 0.87
Pyrophen	29	1.30–6.45	3.09 ± 1.59	14	1.04–95.1	23.7 ± 36.2
*Penicillium*						
Aurantine	9	1.25–16.4	5.41 ± 7.33	3	1.38	1.38
Barceloneic acid	87	7.75–2630	316 ± 497	56	13.3–2220	1060 ± 2230
Citreorosein	87	2.77–104.6	27.04 ± 25.7	59	1.91–79.544	17.61 ± 23.2
Brefeldin A	11	41–1150	786 ± 528	3	289	289 ± 0.00
Citreohybridinol	69	1.19–22,600	35.9 ± 36.6	17	9.38–114	1630 ± 5050
Curvularin	100	9.77–5780	403.7 ± 755	100	4.48–3080	754.7 ± 1080
Dechlorogriseofulvin	16	2.90–53.7	11.26 ± 16.52	22	1.6–18.6	6.60 ± 5.92
Dehydrocurvularin	18	104–758	588 ± 459	14	138–1340	247 ± 157
Dichlordiaportin	93	5.63–482	70 ± 104	72	4.20–435	147 ± 170
Griseofulvin	62	0.58–14.1	4.50 ± 6.59	31	0.55–13.3	4.92 ± 4.65
Herquline A	29	0.52–1.88	1.97 ± 1.41	N/D	N/D	N/D
Hydroxycurvularin	64	41.1–697	43.3 ± 37.4	25	11.8–137	132 ± 133
Pinselin	49	1.28–25.5	6.79 ± 7.46	36	0.81–26.5	6.29 ± 6.12
Quinolactacin A	22	0.87–84.1	18.9 ± 28.8	11	0.53–68.6	9.20 ± 18.3
Thielavin B	40	1–3.8	3.86 ± 3.52	19	0.40–7.70	0.87 ± 0.86
*Fusarium*						
Aminodimethyloctadecanol	7	1570–2420	1877 ± 387	N/D	N/D	N/D
Antibiotic Y	11	34.2–103.4	64.8 ± 29.2	17	41.46–616	287.8 ± 236.3
Aurofusarin *	98	10.7–4230	672 ± 791	86	10.5–89	669 ± 1644
Bikaverin	100	18.7–2390	618 ± 645	50	2.43–3920	360 ± 874
Epiequisetin	N/D	N/D	N/D	11	0.84–30.4	12.9 ± 13.6
Equisetin	31	0.23–5.40	1.41 ± 1.33	67	0.79–103	22.3 ± 24.4
Fuscofusarin *	47	4.03–2720	141,000 ± 94,800	53	19,000–1,400,000	190,000 ± 322,000
Sambucinol	N/D	N/D	N/D	3	27.86	27.86
Siccanol *	80	55,500–1,510,000	714,000 ± 747,000	69	52,000–14,300,000	1,210,000 ± 2,780,000
*Alternaria*						
Altersetin	87	3.23–381	54.09 ± 100.87	61	5.09–618	55.6 ± 132.18
Altersolanol	42	428.6–21,300	4710.70 ± 6568.10	25	670.5–14100	3725.3 ± 4333.3
Macrosporin	87	0.79–154	24.58 ± 30.28	78	1.58–84.1	25.6 ± 21.2
Pyrenophorol	51	3.57–30.6	11.8 ± 8.6	25	3.50–31.3	11.4 ± 9.04

For metabolites indicated by * no quantitative standards were available, therefore numbers denote LC-MS/MS peak area in order to enable relative comparison. N/D = Not detected.

**Table 3:** Metabolites produced by unspecified, uncommon fungal genera and other organisms quantified in sorghum malts.

Compounds	Origin	*Omalodu* Malts *n* = 45				*Otombo* Malts *n* = 36	
		Prevalence (%)	Range [(μg/kg) or Peak Area^2^]	Average [(μg/kg) or Peak Area^2^]	Prevalence (%)	Range [(μg/kg) or Peak Area^2^]	Average [(μg/kg) or Peak Area^2^]
Abscisic acid	*Botrytis* and plants	2	2380	2380	N/D	N/D	N/D
Antibiotic PF 1052	*Phoma*	4	27.7–274	90 ± 57.8	5	9.63–46.8	28.2 ± 18.6
Asperglaucide	Unspecific	24	0.13–17.4	2.67 ± 4.37	19	0.12–1.37	0.64 ± 0.45
Bassianolide	*Cladosporium*	69	0.10–5.29	0.67 ± 0.89	17	0.10–0.33	0.21 ± 0.09
Brevianamide F	Fungi and bacterial	100	37.6–427	144 ± 92.6	94	23.09–312	90.1 ± 56.1
Calphostin	*Metarhizium*	2	14.23	14.23	17	11.10–50.1	20.5 ± 14.9
Chloramphenicol	Bacterial	84	11.4–3173.3	484.9 ± 745.5	28	0.14–0.90	0.44 ± 0.26
Cyclo (l-Pro-l-Tyr)	Unspecific	100	48.2–48,200	121 ± 33.8	94	32.5–165	75.6 ± 31.5
Cyclo (l-Pro-l-Val)	Unspecific	100	42.7–345	456 ± 158	100	2.13–420	121 ± 86.4
Destruxin A	*Metarhizium*	29	0.25–2.06	0.73 ± 0.30	22	0.29–12.7	2.39 ± 3.90
Destruxin-Ed Derivative	*Metarhizium*	7	0.83–7.1	3.32 ± 2.72	8	0.81–13.4	5.59 ± 5.56
Dihydroxymellein	Unspecific	N/D	N/D	N/D	8	31.2–107	68.2 ± 31.3
Fellutanine A	Unspecific	82	7.14–25.5	16.1 ± 5.79	75	3.39–22.6	11.2 ± 4.46
Heptelidic acid	*Phoma*	7	33.2–87.09	53.9 ± 28.9	6	37.7–60.7	49.9 ± 16.2
Monactin	Bacterial	40	0.27–5.16	1.50 ± 1.26	19	0.45–1.83	0.89 ± 0.54
Monocerin	Unspecific	100	293–1120	139 ± 226	89	1.28–560	47.5 ± 100
Orsellinic acid	Unspecific	98	115–21,000	4080 ± 4748	47	1090–17,000	3780 ± 3690
Phomalactone	*Trichoderma*	18	1.12–7.83	1.65 ± 1.21	8	2.05–5.49	3.52 ± 1.77
Rugulusovin	Unspecific	100	27.7–157	108 ± 47.6	94	5.40–254.3	47.8 ± 51.4
Tryptophol	Unspecific	N/D	N/D	N/D	81	16.7–352	110 ± 80.5
Skyrin	Unspecific	91	0.50–23.9	0.69 ± 0.42	47	0.38–2.08	0.73 ± 0.42
Siccanin	*Helmintosporum*	98	3.19–9.95	7.01 ± 1.45	14	3.73–10.6	6.11 ± 2.67

N/D = Not detected.

**Table 4:** Performance characteristics of the method for some metabolites quantified in sorghum malts.

Compounds	*Omalodu* and *Otombo* Malts		*Omalodu* Malts		*Otombo* Malts	
	LOD (ng/g)	LOQ (ng/g)	Apparent Recovery (%)	RSD (%) (*n* = 3)	Apparent Recovery (%)	RSD (%) (*n* = 3)
3-Nitropropionic acid	0.71	2.4	147	47.13	219	36.75
Abscisic acid	15	50	192	31.79	N/D	N/D
Aflatoxin B1	0.17	0.57	40	1.52	40	3.11
Aflatoxin B2	0.04	0.13	40	1.7	50	2.88
Aflatoxin G1	0.1	0.35	46	1.3	45	2.63
Agroclavine	0.1	0.32	81	3.85	92	8.14
Alternariol	0.1	0.32	45	9.17	40	6.97
Alternariolmethylether	0.11	0.38	75	10.37	64	2.16
Altersetin	0.89	3	163	17.35	158	16.75
Altersolanol	126	421	248	113.25	125	73.16
Antibiotic PF 1052	2.5	8.2	120	1.88	176	30.81
Antibiotic Y	6.9	23	154	10.67	154	30.57
Asperfuran	3.7	12	94	8.91	106	0
Asperglaucide	0.03	0.12	66	6.52	78	2.31
Asterric acid	0.15	0.5	186	12.25	208	12.17
Aurantine	0.26	0.85	62	5.95	58	1.85
Averufin	0.02	0.07	N/D	N/D	51	3.9
Barceloneic acid	0.55	1.8	439	252.56	381	180.88
Bassianolide	0.02	0.08	75	16.97	82	12.94
Beauvericin	0.06	0.2	82	28.57	81	0
Bikaverin	0.46	1.5	82	0	129	0
Bis (methylthio)gliotoxin	0.86	2.9	60	2.36	65	1.9
Brefeldin A	20	66	129	35.89	87	10.58
Brevianamid F	0.35	1.2	110	5.75	111	0
Calphostin	3.4	11	115	15.42	127	11.35
Chanoclavin	0.02	0.07	82	6.38	78	6.98
Chloramphenicol	0.03	0.09	84	3.53	58	18.4
Citreohybridinol	0.09	0.31	77	8.18	91	21.22
Citreorosein	0.74	2.5	66	5.85	64	15.5
Curvularin	0.36	1.2	111	0	110	0
Cyclo (l-Pro-l-Tyr)	8.5	28	74	5.16	99	28.37
Cyclo (l-Pro-l-Val)	1.2	3.9	268	16.23	255	0
Cyclopiazonic acid	15	50	137	13.65	128	10.12
Cytochalasin E	0.43	1.4	99	7.21	113	0
Dechlorogriseofulvin	0.43	1.4	111	11.61	93	0.36
Dehydrocurvularin	0.96	3.2	87	7.54	60	0
Demethylsulochrin	0.58	1.9	118	11.63	120	0
Deoxynortryptoquivalin	0.62	2.1	106	30.01	77	0
Deoxytryptoquivaline A	0.27	0.89	87	11.75	76	0
Destruxin A	0.07	0.24	68	2.19	70	0.82
Destruxin-ed derivative	0.66	2.2	74	2	78	0.99
Dichlordiaportin	0.64	2.1	110	15.2	130	0
Dihydrocitrinone	0.77	2.6	121	6.31	143	0
Dihydroxymellein	0.51	1.7	N/D	N/D	136	16.66
Elymoclavine	0.18	0.59	65	3.54	76	8.55
Emodin	0.06	0.2	104	11.21	104	3.59
Epiequisetin	0.1	0.32	N/D	N/D	132	14.5
Equisetin	0.1	0.33	138	16.46	148	10.02
Fellutanine A	0.48	1.6	102	10.17	113	3.86
Festuclavine	0.02	0.07	76	3.92	86	17
Flavoglaucin	0.03	0.1	149	14.27	127	0
Fumagillin	9	30	52	7.91	59	16.34
Fumigaclavine C	0.83	2.8	87	3.06	95	4.61
Fumiquinazolin A	0.18	0.59	84	5.57	93	11.4
Fumiquinazolin D	0.27	0.9	74	1.22	97	19.86
Fumitremorgin C	0.19	0.62	42	4.36	42	8.33
Fumonisin B1	2.4	8	70	11.34	77	6.52
Fumonisin B2	1.7	5.6	74	10	83	4.89
Fumonisin B3	5.8	19	74	10.56	80	6.64
Gliotoxin	0.91	3	42	8.79	47	11.15
Griseofulvin	0.14	0.46	71	1.57	70	0.33
Helvolic acid	2.1	6.9	119	2.97	138	15.56
Heptelidic acid	8.7	29	135	23.55	112	1.27
Herquline A	0.06	0.22	66	5.15	53	8.88
Hydroxycurvularin	0.94	3.1	108	20.09	90	13.67
Iso-rhodoptilometrin	0.03	0.09	55	6.08	60	5.26
Kojic acid	20	68	288	152.08	1013	426.7
Macrosporin	0.13	0.44	55	1.04	57	11.02
Moniliformin	1	3.4	106	13	139	14.96
Monocerin	0.06	0.19	85	0	89	2.58
Patulin	11	36	71	13.25	55	0
Phenopyrrozin	0.31	1	96	15.14	164	28.84
Phomalactone	0.61	2	64	5.16	60	5.98
Pinselin	0.6	2	65	2.01	70	3.84
Pseurotin A	1.5	5	83	2.38	92	4.95
Pyrenophorol	0.96	3.2	73	4.2	77	8.29
Quinolactacin A	0.01	0.03	62	5.24	72	8.77
Rugulusovin	0.45	1.5	81	11.08	136	34.61
Sambucinol	4.5	15	87	7.33	92	17.49
Siccanin	0.93	3.1	69	1.87	76	6.35
Skyrin	0.08	0.26	94	13.05	105	5.66
Sterigmatocystin	0.06	0.19	50	2.01	57	3.15
Tenuazonic acid	30	100	321	0	275	0
Thielavin B	0.29	0.98	44	6.65	58	3.08
Trypacidin	0.09	0.29	53	17.64	N/D	N/D
Tryprostatin B	1.5	5.1	43	2.3	38	3.85
Tryptophol	3.5	12	N/D	N/D	60	5.95
Tryptoquivaline A	0.48	1.6	74	1.91	84	0
Tryptoquivaline F	0.67	2.2	79	10.81	104	23
Versicolorin C	0.13	0.45	94	26.14	53	15.77

N/D = Not detected.

## References

[1] Roger D.D. (2011). Deoxynivalenol (DON) and fumonisins B_1_ (FB_1_) in artisanal Sorghum opaque beer brewed in north Cameroon. Afr. J. Microbiol. Res..

[2] Dicko M.H., Gruppen H., Traoré A.S., Voragen A.G., Van Berkel W.J. (2006). Sorghum grain as human food in Africa: Relevance of content of starch and amylase activities. Afr. J. Biotechnol..

[3] Misihairabgwi J., Cheikhyoussef A. (2017). Traditional fermented foods and beverages of Namibia. J. Ethn. Foods.

[4] Adebiyi J.A., Obadina A.O., Adebo O.A., Kayitesi E. (2018). Fermented and malted millet products in Africa: Expedition from traditional/ethnic foods to industrial value-added products. Crit. Rev. Food Sci. Nutr..

[5] Onesmo N.M. (2011). Effects of Malting and Fermentation on the Composition and Functionality of Sorghum Flour. Master’s Thesis.

[6] Tangni E.K., Larondelle Y. Malts, moulds and mycotoxins. Bacteria, Yeasts and Moulds in Malting and Brewing. Proceedings of the Xth Symposium “Chair J. de Clerck”.

[7] Wagacha J.M., Muthomi J.W. (2008). Mycotoxin problem in Africa: Current status, implications to food safety and health and possible management strategies. Int. J. Food Microbiol..

[8] Hajslova J., Zachariasova M., Cajka T. (2011). Analysis of multiple mycotoxins in food. Methods Mol. Biol..

[9] Reddy K.R.N., Salleh B., Saad B., Abbas H.K., Abel C.A., Shier W.T. (2010). An overview of mycotoxin contamination in foods and its implications for human health. Toxin Rev..

[10] Bennett J., Klich M. (2003). Mycotoxins. Clin. Microbiol. Rev..

[11] Richard J.L. (2007). Some major mycotoxins and their mycotoxicoses—An overview. Int. J. Food Microbial..

[12] Schatzmayr G., Streit E. (2013). Global occurrence of mycotoxins in the food and feed chain: Facts and figures. World Mycotoxin J..

[13] Gruber-Dorninger C., Novak B., Nagl V., Berthiller F. (2016). Emerging mycotoxins: Beyond traditionally determined food contaminants. J. Agric. Food Chem..

[14] Vaclavikova M., Malachova A., Veprikova Z., Dzuman Z., Zachariasova M., Hajslova J. (2013). ‘Emerging’mycotoxins in cereals processing chains: Changes of enniatins during beer and bread making. Food Chem..

[15] Kovalsky P., Kos G., Nährer K., Schwab C., Jenkins T., Schatzmayr G., Sulyok M., Krska R. (2016). Co-occurrence of regulated; masked and emerging mycotoxins and secondary metabolites in finished feed and maize—An extensive survey. Toxins.

[16] Lee H.J., Ryu D. (2017). Worldwide occurrence of mycotoxins in cereals and cereal-derived food products: Public health perspectives of their co-occurrence. J. Agric. Food Chem..

[17] Odhav B., Naicker V. (2002). Mycotoxins in South African traditionally brewed beers. Food Addit. Contam..

[18] Gamanya R., Sibanda L. (2001). Survey of *Fusarium moniliforme* (*F. verticillioides*) and production of fumonisin B_1_ in cereal grains and oilseeds in Zimbabwe. Int. J. Food Microbiol..

[19] Nkwe D.O., Taylor J.E., Siame B.A. (2005). Fungi, Aflatoxins, Fumonisin B_l_ and Zearalenone Contaminating Sorghum-based Traditional Malt, Wort and Beer in Botswana. Mycopathologia.

[20] Misihairabgwi J.M., Ishola A., Quaye I., Sulyok M., Krska R. (2018). Diversity and fate of fungal metabolites during the preparation of *oshikundu*, a Namibian traditional fermented beverage. World Mycotoxin J..

[21] Matumba L., Monjerezi M., Khonga E.B., Lakudzala D.D. (2011). Aflatoxins in sorghum; sorghum malt and traditional opaque beer in southern Malawi. Food Control.

[22] Garba M.H., Makun H.A., Jigam A.A., Muhammad H.L., Patrick B.N. (2017). Incidence and toxigenicity of fungi contaminating sorghum from Nigeria. World J. Microbiol..

[23] Chala A., Taye W., Ayalew A., Krska R., Sulyok M., Logrieco A. (2014). Multi-mycotoxin analysis of sorghum (*Sorghum bicolor* L. Moench) and finger millet (*Eleusine coracana* L. Garten) from Ethiopia. Food Control.

[24] Alshannaq A., Yu J.H. (2017). Occurrence, toxicity, and analysis of major mycotoxins in food. Int. J. Environ. Res..

[25] Marin S., Ramos A.J., Cano-Sancho G., Sanchis V. (2013). Mycotoxins: Occurrence; toxicology; and exposure assessment. Food Chem. Toxicol..

[26] European Commission (EC) (2006). Commission Regulation (EC) No 1881/2006 of 19 December 2006 setting maximum levels for certain contaminants in foodstuffs. Off. J. Eur. Union.

[27] Bationo J.F., Nikiéma P.A., Koudougou K., Ouédraogo M., Bazié S.R., Sanou E., Barro N. (2015). Assessment of aflatoxin B_1_ and ochratoxin A levels in sorghum malts and beer in Ouagadougou. Afr. J. Food Sci..

[28] Zbyňovská K., Petruška P., Kalafová A., Capcarová M. (2016). Patulin-a Contaminant of Food and Feed: A review. Acta Fytotech. Zootechn..

[29] Assunção R., Martins C., Dupont D., Alvito P. (2016). Patulin and ochratoxin A co-occurrence and their bioaccessibility in processed cereal-based foods: A contribution for Portuguese children risk assessment. Food Chem. Toxicol..

[30] Ioi J.D., Zhou T., Tsao R.F., Marcone M. (2017). Mitigation of patulin in fresh and processed foods and beverages. Toxins.

[31] Glaser N., Stopper H. (2012). Patulin: Mechanism of genotoxicity. Food Chem. Toxicol..

[32] International Agency for Research on Cancer (1976). IARC Monographs on the Evaluation of Carcinogenic Risks to Human: Some Naturally Occurring Substances.

[33] Gao W., Jiang L., Ge L., Chen M., Geng C., Yang G., Li Q., Ji F., Yan Q., Zou Y. (2015). Sterigmatocystin-induced oxidative DNA damage in human liver-derived cell line through lysosomal damage. Toxicol. In Vitro.

[34] Zouaoui N., Mallebrera B., Berrada H., Abid-Essefi S., Bacha H., Ruiz M.J. (2016). Cytotoxic effects induced by patulin., sterigmatocystin and beauvericin on CHO–K1 cells. Food Chem. Toxicol..

[35] Liu Y., Du M., Zhang G. (2014). Proapoptotic activity of aflatoxin B_1_ and sterigmatocystin in HepG2 cells. Toxicol. Rep..

[36] Lee W.T., Chang C. (2004). Magnetic resonance imaging and spectroscopy in assessing 3-nitropropionic acid-induced brain lesions: An animal model of Huntington’s disease. Prog. Neurobiol..

[37] Carvajal-Moreno M. (2015). Mycotoxins that affect the human cardiovascular system. Pharm. Anal. Acta.

[38] Li Y.C., Ledoux D.R., Bermudez A.J., Fritsche K.L., Rottinghaus G.E. (2000). The individual and combined effects of fumonisin B_1_ and moniliformin on performance and selected immune parameters in turkey poults. Poult. Sci..

[39] Huang P.H., Huang C.Y., Chen M.C., Lee Y.T., Yue C.H., Wang H.Y., Lin H. (2013). Emodin and aloe-emodin suppress breast cancer cell proliferation through ERα inhibition. Evid. Based Complement. Altern. Med..

[40] Wang Q.J., Cai X.B., Liu M.H., Hu H., Tan X.J., Jing X.B. (2010). Apoptosis induced by emodin is associated with alterations of intracellular acidification and reactive oxygen species in EC-109 cells. Int. J. Biochem. Cell Biol..

[41] Liu Z., Wei F., Chen L.J., Xiong H.R., Liu Y.Y., Luo F., Hou W., Xiao H., Yang Z.Q. (2013). In Vitro and in Vivo Studies of the Inhibitory Effects of Emodin Isolated from Polygonum cuspidatum on Coxsackievirus B4. Molecules.

[42] Hsiang C.Y., Ho T.Y. (2008). Emodin is a novel alkaline nuclease inhibitor that suppresses herpes simplex virus type 1 yields in cell cultures. Br. J. Pharmacol..

[43] Chukwujekwu J.C., Coombes P.H., Mulholland D.A., Van Staden J. (2006). Emodin, an antibacterial anthraquinone from the roots of *Cassia occidentalis*. S. Afr. J. Bot..

[44] Jow G.M., Chou C.J., Chen B.F., Tsai J.H. (2004). Beauvericin induces cytotoxic effects in human acute lymphoblastic leukaemia cells through cytochrome c release, caspase 3 activation: The causative role of calcium. Cancer Lett..

[45] Smith E.R., Fredrickson T.N., Hadidian Z. (1968). Toxic effects of the sodium and the N, N′-dibenzylethylenediamine salts of tenuazonic acid (NSC-525816 and NSC-82260). Cancer Chemother. Rep..

[46] Patriarca A., Azcarate M.P., Terminiello L., Pinto V.F. (2007). Mycotoxin production by *Alternaria* strains isolated from Argentinean wheat. Int. J. Food Microbiol..

[47] Giambrone J.J., Davis N.D., Diener U.L. (1978). Effect of tenuazonic acid on young chickens. Poult. Sci..

[48] Fleck S.C., Burkhardt B., Pfeiffer E., Metzler M. (2012). *Alternaria* toxins: Altertoxin II is a much stronger mutagen and DNA strand breaking mycotoxin than alternariol and its methyl ether in cultured mammalian cells. Toxicol. Lett..

[49] Bandyopadhyay R., Butler D.R., Chandrashekar A., Reddy R.K., Navi S.S. (2000). Biology, epidemiology, and management of sorghum grain mould. Technical and Institutional Options for Sorghum Grain Mould Management: Proceedings of an International Consultation.

[50] Bandyopadhyay R., Frederickson D.E., McLaren N.W., Odvody G.N., Riley M.J. (1998). Ergot: A new disease threat to sorghum in the Americas and Australia. Plant Dis..

[51] Kwon-Chung K.J., Sugui J.A. (2009). What do we know about the role of gliotoxin in the pathobiology of *Aspergillus fumigatus*?. Med. Mycol..

[52] Domingo M.P., Colmenarejo C., Martínez-Lostao L., Müllbacher A., Jarne C., Revillo M.J., Delgado P., Roc L., Meis J.F., Rezusta A. (2012). Bis (methyl) gliotoxin proves to be a more stable and reliable marker for invasive aspergillosis than gliotoxin and suitable for use in diagnosis. Diagn. Microbiol. Infect. Dis..

[53] Ostry V., Toman J., Grosse Y., Malir F. (2018). Cyclopiazonic acid: 50th anniversary of its discovery. World Mycotoxin J..

[54] Stierle A.C., Cardellina J.H., Strobel G.A. (1988). Maculosin. A host-specific phytotoxin for spotted knapweed from *Alternaria alternata*. Proc. Natl. Acad. Sci. USA.

[55] Puopolo G., Cimmino A., Palmieri M.C., Giovannini O., Evidente A., Pertot I. (2014). *Lysobacter capsici* AZ78 produces cyclo (l-Pro-l-Tyr), a 2, 5-diketopiperazine with toxic activity against sporangia of *P. phytophthora infestans* and *P. lasmopara viticola*. J. Appl. Microbiol..

[56] Capon R.J., Stewart M., Ratnayake R., Lacey E., Gill J.H. (2007). Citromycetins and bilains A-C: New aromatic polyketides and diketopiperazines from Australian marine-derived and terrestrial *Penicillium* spp.. J. Nat. Prod..

[57] Borthwick A.D. (2012). 2, 5-Diketopiperazines: Synthesis, reactions, medicinal chemistry and bioactive natural products. Chem. Rev..

[58] Kozlovskiĭ A.G., Vinokurova N.G., Adanin V.M. (2000). Diketopiperazine alkaloids from the fungus *Penicillium piscarium* Westling. Prikl. Biokhim. Mikrobiol..

[59] Cornford E.M., Bocash W.D., Braun L.D., Crane P.D., Oldendorf W.H., MacInnis A.J. (1979). Rapid distribution of tryptophol (3-indole ethanol) to the brain and other tissues. J. Clin. Investig..

[60] Stadler D., Sulyok M., Schuhmacher R., Berthiller F., Krska R. (2018). The contribution of lot-to-lot variation to the measurement uncertainty of an LC-MS-based multi-mycotoxin assay. Anal. Bioanal. Chem..

[61] Malachová A., Sulyok M., Beltrán E., Berthiller F., Krska R. (2014). Optimization and validation of a quantitative liquid chromatography-tandem mass spectrometric method covering 295 bacterial and fungal metabolites including all regulated mycotoxins in four model food matrices. J. Chromatogr. A.

[62] European Union Commission (2002). Decision 2002/657/EC of 12 August 2002 implementing Council Directive 96/23/EC concerning the performance of analytical methods and the interpretation of results. Off. J. Eur. Union.

[63] Magnusson B., Örnemark U. (2014). Eurachem Guide: The Fitness for Purpose of Analytical Methods—A Laboratory Guide to Method Validation and Related Topics.

